# Ex Vivo Lung Perfusion with K(ATP) Channel Modulators Antagonize Ischemia Reperfusion Injury

**DOI:** 10.3390/cells10092296

**Published:** 2021-09-03

**Authors:** Stephan Arni, Tatsuo Maeyashiki, Tsogyal Latshang, Isabelle Opitz, Ilhan Inci

**Affiliations:** 1 Department of Thoracic Surgery, University Hospital Zürich, 8091 Zürich, Switzerland; stephan.arni@usz.ch (S.A.); tmaeya@juntendo.ac.jp (T.M.); Isabelle.Schmitt-Opitz@usz.ch (I.O.); 2 Department of Pneumology, Kantonsspital Graubünden, 7000 Chur, Switzerland; tsogyal.latshang@ksgr.ch

**Keywords:** ex vivo lung perfusion, ATP sensitive potassium channels, donation after circulatory death, lung transplantation

## Abstract

Ex vivo lung perfusion (EVLP) has been implemented to increase the number of donor lungs available for transplantation. The use of K(ATP) channel modulators during EVLP experiments may protect against lung ischemia-reperfusion injury and may inhibit the formation of reactive oxygen species. In a rat model of donation after circulatory death with 2 h warm ischemic time, we evaluated rat lungs for a 4-hour time in EVLP containing either mitochondrial-specific or plasma membrane and/or sarcolemmal-specific forms of K(ATP) channel modulators. Lung physiological data were recorded, and metabolic parameters were assessed. When compared to the control group, in the EVLP performed with diazoxide or 5-hydroxydecanoic acid (5-HD) we recorded significantly lower pulmonary vascular resistance and only in the diazoxide group recorded significant lung weight loss. In the perfusate of the 5-HD group, interleukin-1β and interleukin-1α were significantly lower when compared to the control group. Perfusate levels of calcium ions were significantly higher in both 5-HD and cromakalim groups, whereas the levels of calcium, potassium, chlorine and lactate were reduced in the diazoxide group, although not significantly when compared to the control. The use of a diazoxide mitochondrial-specific K(ATP) channel opener during EVLP improved lung physiological and metabolic parameters and reduced edema.

## 1. Introduction

Currently, lung transplantation is the accepted treatment option for patients in end-stage lung disease who are failing maximal medical therapy. The most common indications for lung transplantation are advanced chronic obstructive pulmonary disease (40% of total worldwide lung transplantations), interstitial lung disease, which carries the worst prognosis among the common disease indications for lung transplantation, cystic fibrosis, emphysema due to alpha-1 antitrypsin deficiency, and pulmonary arterial hypertension. There is an expanding pool of combined heart-lung, liver-lung and kidney-lung transplant candidates although less frequently indicated [1]. The constant increase in the number of centers worldwide performing lung transplantations has a tremendous impact for lung transplant candidates in terms of morbidity and mortality [2]. Nevertheless, the number of discarded organs is increasing constantly, primarily as a consequence of the increasing average age of the donor pool. At the same time, waiting list mortality is still an issue among lung transplant candidates, even after three decades of success stories in the field [2], with the demand in the US for donor lungs still outpacing the current supply at an estimated rate of 17.2 pre-transplant deaths per 100 waitlist-years [2]. Severe primary graft dysfunction is the primary cause of lung transplantation failure with early mortality, and has important financial consequences for the caring institutions, causing prolonged stays in intensive care units [3]. Clinically described as primary dysfunction, the disease is the consequence of ischemia-reperfusion (I/R) injuries characterized by sterile inflammation, endothelial cell dysfunction, oedema, increased pulmonary vascular resistance (PVR) and impaired oxygen exchange [4]. Novel methods to increase the number of donor lungs available for transplantation have been implemented to overcome this shortage, such as the application of extended criteria (marginal) donor lungs, donation after circulatory death donors (DCD) [5], living donor lobar lung transplantation, and ex vivo lung perfusion (EVLP) for the re-evaluation of questionable donor lungs [6,7]. However, the demand for donor lungs continues to be outpaced by an ever-growing waitlist [2]. EVLP provides potential for the re-evaluation, treatment, and repair of donor lungs that are not suitable for transplantation. I/R injury is complex and multifactorial. One of its mechanisms is the endothelial activation and upregulation of the adhesion molecules [8,9], leading to severe damages to the cellular structure. It has been hypothesized that, after stopping the flow in the lung endothelium and during the graft preservation cold ischemic time, mechanosignaling cascades initiate an inhibiting response of the sodium Na^+^-K^+^ ATPase and various K^+^ channels. This biochemical cascade leads to Na^+^ influx with persistent cell membrane depolarization due to a decrease in intracellular K^+^ [10,11], and finally leads to an increase in reactive oxygen species (ROS) in the mitochondria [12] and to enhanced activity of ROS-producing enzymes [13]. Under physiological conditions, active K^+^ channels are responsible for antagonizing Na^+^ influx and establishing membrane hyperpolarization (i.e., favoring hyperpolarization). However, I/R injury is associated with the inhibition of various K^+^ channels, including Na^+^-K^+^ ATPase and Ca^++^-sensitive K^+^ channels. This cascade is characterized by a decrease in intracellular K^+^ that results in inflammasomes activation with the formation of the NLR family pyrin domain containing 3 (NLRP3) machinery, which cleaves pro-caspase-1 to generate caspase-1. Caspase-1 drives the processing of pro-IL-1β to IL-1β as well as that of pro-IL-18 to IL-18, with both of these cytokines being able to activate IL-6 and other pro-inflammatory cytokines [14,15].

In this study, we used two mitochondria-specific K(ATP) channel modulators, the channel opener diazoxide (DZ) and the channel blocker 5-hydroxydecanoic acid (5-HD). We also evaluated two plasma membrane and/or sarcolemmal-specific forms of K(ATP) channel modulators, the channel opener cromakalim (CK) [16] and the channel blocker glybenclamide (GL) [17]. So far, there have been no reports testing K(ATP) channel modulators during EVLP with warm ischemic DCD donor lungs. Therefore, it remains unclear whether any of these K(ATP) modulators, which have already been used in the context of I/R injury in ischemic organ transplantation settings but failed to pass in the clinic, may be of interest during ex vivo re-evaluation of questionable donor lungs. Moreover, the optimal time to administrate those agents (either before or during the cold ischemic storage and/or during EVLP) should be defined to demonstrate the potential protective effect of these agents against I/R injury in lung transplantation [13].

## 2. Materials and Methods

### 2.1. Animals

The Veterinarian committee Kanton Zurich approved the animal use for this study (ZH080/17). Male Sprague Dawley rats (Janvier Labs, Le Genest Saint-Isle, France) weighing 280–360 g were maintained in a pathogen-free environment and received adequate care according to “Guide for the Care and Use of Laboratory Animals: Eighth Edition” [18]. This study was carried out in compliance with the ARRIVE guidelines.

### 2.2. Surgical Techniques for Procurment of Donation after Circulatory Death Donor Lung and EVLP Model

All rats were anaesthetized with 2–3% (*vol*/*vol*) isoflurane in O_2_. After being fully anesthetized, the animals were orotracheally intubated with a 14-gauge intravenous catheter and underwent mechanical ventilation with a rodent ventilator (Harvard Apparatus, Inc., Model Ventelite, Holliston, MA, USA). Rats were administered volume control ventilation at a respiratory rate of 60 breaths/min with a tidal volume of 10 mL/kg, an inspired oxygen fraction (FiO_2_) of 1 and a positive end-expiratory pressure (PEEP) set at 3 cmH_2_O. Following laparotomy and sternotomy, we injected 300 IU heparin into the inferior vena cava and sacrificed the rats by clamping the ascending aorta. DCD donor lungs were collected as reported [19], and somewhat mimicked DCD clinical category. After 2 h of warm ischemic time in situ, we inserted the perfusion cannula (Hugo Sachs Elektronik Harvard Apparatus, March-Hugstetten, Germany) in the pulmonary artery and the left atrium. The lungs were flushed at a perfusion pressure of 20 cm H_2_O with 20 mL of cold low-potassium dextran solution (Perfadex plus, XVIVO, Göteborg, Sweden) through the pulmonary artery cannula. Finally, the heart lung block was weighed.

### 2.3. EVLP Procedure with K(ATP) Channel Modulators and Physiological Variables

The DCD lungs were perfused for 4 h in an Isolated Perfused Lung system for rats and guinea pigs (IPL-2, Hugo Sachs Elektronik Harvard Apparatus, March-Hugstetten, Germany) under positive pressure ventilation and at a standard normothermic temperature of 37 °C. As a perfusate, we selected 125 mL of the acellular Steen solution (Steen solution, XVIVO, Göteborg, Sweden) supplemented with 300 IU sodium heparin, antibiotic (50 mg meropenem, Labatech Pharma, Meyrin, Switzerland), and methylprednisolone (50 mg Solu-Medrol, Pfizer Inc., New York, NY, USA). We tested in this EVLP setting both mitochondria-specific K(ATP) channel modulators such as the K(ATP) channel opener diazoxide (DZ) and the K(ATP) channel blocker 5-hydroxydecanoic acid (5-HD), or plasma membrane and/or sarcolemmal-specific K(ATP) channel modulators such as the opener cromakalim (CK) and the blocker glybenclamide (GL). All the K(ATP) channel modulators were from Cayman Chemical (Cayman Chemical, Ann Arbor, MI, USA), except for 5-HD, which was obtained from Santa Cruz Biotechnology (Santa Cruz Biotechnology, Dallas, TX, USA). The DZ, GL and CK powder were resuspended in dimethyl sulfoxide (DMSO), whereas 5-HD was resuspended in sterile water. All the aliquoted stocks of the K(ATP) channel modulators were frozen at −20 °C until the experiment. Before starting the 4 h EVLP, an aliquot was thawed and mixed at 20 °C in the Steen solution. The circuit perfusate temperature was gradually increased by using a thermostatic water bath and the targeted temperature of 37 °C was reached after 20 min and was maintained throughout the 4 h EVLP. The left atrium pressure was set at 2–3 cmH_2_O and the automatized IPL-2 controller system maintained the pulmonary arterial pressure (PAP) below 15 cmH_2_O by adjusting the flow. Ventilation with the IPL-2 ventilator (VCM-P, Hugo Sachs Elektronik Harvard Apparatus, March-Hugstetten, Germany) started at a perfusate temperature of 37 °C after 20 min reperfusion time and with 30% of the targeted flow. The fixed tidal volume was at 5 mL/kg, with an inspiratory/expiratory ratio of 1/3, a rate of 30 breaths/min and with a PEEP of 3 cmH_2_O and an inspired oxygen fraction (FiO_2_) of 0.21. Thereafter, the perfusate was deoxygenated with a mixture of 8% CO_2_ and 92% N_2_ using a gas exchange membrane (D-150 hemofilter, Medsulfone, Medica S.p.A., Italy). The 100% targeted flow was calculated as the 20% of a 250 g rat with a 75 mL/min cardiac output. We started lung perfusion with 10% of the targeted flow (1.5 mL/min) for 10 min. The four following 10 min steps in mL/min were 3, 4.5, 7.5, and 12 mL/min. Then, at the 50 min time point, we switched to the maximum flow of 15 mL/min. We recorded all the respiratory parameters with a dedicated software (PULMODYN^®^ HSE software, Hugo Sachs Elektronik Harvard Apparatus, March-Hugstetten, Germany). We monitored PAP, peak airway pressure and airway flow during EVLP. Hourly, and 5 min after switching ventilation with a FiO_2_ of 1, we recorded the dynamic lung compliance (Cdyn), the PVR and sampled the perfusate. At the end of the 4 h EVLP, we performed an additional stress test where the IPL-2 controller system was disabled, allowing the flow to increase over the 100% targeted flow up to the maximum PAP value set at 15 cm H_2_O. After a 5 min stress test, we recorded flow, PVR and Cdyn. For further analysis, an aliquot of the lungs was flash-frozen in liquid nitrogen. All samples were stored at −80 °C until further examination. The five groups of rats were adjusted for equivalent mean body weight (data not shown). We performed sample size calculation using G*power 3 software version 3.3.7 [20]. Six animals per group was required to achieve 90% power to obtain a signal/noise ratio of 2.0. Six EVLP were performed for the control, DZ, 5-HD, and CK groups. For the GL group, since the important parameters (PVR, cDyn, flow) did not reach values at least matching the control group, we decided to not continue with more rats in this group and performed only three EVLP.

### 2.4. Clinical Biochemistry Parameters

We used the Epoc^®^ blood analysis system (Epoc^®^ Blood Analysis System, Siemens Healthineers, Erlangen, Germany) for the pH, concentrations of calcium, potassium, glucose, sodium, and lactate, and the partial oxygen pressure. The change in pO_2_ (ΔpO_2_) was calculated according to the following equation: ΔpO_2_ = (partial pulmonary venous pO_2—_pulmonary arterial pO_2_).

### 2.5. MTT Viability Assay with a Rat Epithelial Cell Line for Non-Toxic Dosage of K(ATP) Modulator Concentrations

As a representative of rat lung cells for this pharmacological analysis, we used the lung epithelial IL2 cell line (ATCC CCL-49). The IL2 cells were cultured in Ham’s F12K (Kaighn’s) medium (Thermo Fisher cat 21127022) with 10% (*v*/*v*) heat-inactivated foetal bovine serum, penicillin and streptomycin (100 ng/mL). The IL2 cells at a concentration of 0.6 × 10^5^ cells/mL were left to adhere to a 96-well plate and maintained for 24 h at 37 °C in a 5% CO_2_ humidified incubator. The IL2 cells were treated with the medium alone or with two-fold serial dilutions of the K(ATP) channel modulators starting with the highest concentration at 250 µM for DZ, 5 HD, GL and at 25 µM for CK. After 24 h of incubation, each well was then treated with 20 μL of the tetrazolium dye MTT (3-(4,5-dethylthiazol-2-yl)-2,5-diphenyltetrazolium bromide) solution, and the cells were then resuspended in the wells. After 4 h, the supernatants were removed and 100 μL of DMSO was added to each well to dissolve the precipitate. The cell viability after exposure to an increasing concentration of K(ATP) channel modulators was estimated by measuring absorbance at 570 nm using a Cytation 5 plate reader (BioTek Instruments, Inc., Winooski, VT, USA). The cell viability percentage was calculated based on the absorbance ratio between cell cultures treated with the different K(ATP) channel modulators and the untreated control multiplied by 100 as a representation of cell viability (percentage of control, %) from at least three separate experiments that were performed in duplicate or triplicate. The final concentrations of each K(ATP) modulator used in the EVLP perfusate were selected as follows; DZ, 5-HD and GL were at 100 µM, whereas CK was at 10 µM.

### 2.6. Cytokines, Chemokines and Mediator of Wound Healing

The perfusates collected after 4 h EVLP were flash frozen in liquid nitrogen and stored at −80 °C. We assayed 50 µL of perfusate for cytokines, chemokines and mediators of wound healing levels with a rat cytokine/chemokine panel (Milliplex^®^ Magnetic Bead Panel,; Millipore, Billerica, MA, USA) for simultaneous analysis of interleukins (IL) such as IL-1α, IL-1β, IL-6, IL-18 and chemokines such as monocyte chemoattractant protein 1 (MCP-1), macrophage inflammatory protein-2 (MIP2), macrophage inflammatory protein-1α (MIP-1α), and GRO/KC/CINC-1, and of the growth factor mediator of wound healing VEGF, according to the manufacturer’s instructions.

### 2.7. Estimates of ATP Content, Myeloperoxidase Activity and Carbonyl Protein Content in Lung Tissues

Frozen lung tissue (25 mg) was powdered on dry ice and homogenized in 0.5 mL of 0.5% trichloroacetic acid. We centrifuged the lysates for 2 min at 4 °C and 8000 rpm to separate cleared supernatant from insoluble cell debris. The sample supernatants were buffered with 10× concentrated Tris-acetate buffer containing 10 µL of 0.002% xylenol blue as pH indicator. We used an ATP assay kit (Enliten, Promega, Madison, WI, USA) to estimate the ATP concentration in the supernatant by measuring in the luminescence channel of a Cytation 5 plate reader (BioTek Instruments, Inc., Winooski, VT, USA). The results are expressed in nanomolar ATP per 25 milligram of lung tissue. Tissue lysate extracted from the powdered lung tissue was also analysed using a myeloperoxidase MPO activity assay (OxiSelect™ Myeloperoxidase Chlorination Activity Assay, Cell Biolabs, San Diego, CA, USA) and according to manufacturer’s instructions. The results are expressed in microUnit per milliliter of lung tissue lysate. We used an ELISA-based carbonyl assay (OxiSelect™ Protein Carbonyl Elisa kit, Cell Biolabs, San Diego, CA, USA) to determine the protein accumulation of carbonyl modification in powdered lung tissue lysate according to the manufacturer’s instructions. The results are expressed in nM/mL of lung tissue lysate.

### 2.8. TUNEL Staining and Analysis

A terminal deoxynucleotidyl transferase dUTP nick end labeling (TUNEL) assay was performed on Leica Bond RX using Refine AP-Kit (Bond Polymer Refine Detection kit, cat#DS9800, Leica Microsystems, Inc., Newcastle, UK) including all the buffer-solutions from Leica Microsystems and processed according to the manufacturer guidelines. The antigen retrieval was performed with an enzyme pretreatment kit (Bond Enzyme Pretreatment kit, cat#AR9551, Leica Microsystems Inc., Newcastle, UK) for 25 min at 37 °C. Paraffin slides were dewaxed, pretreated and incubated with mouse anti-DIG FITC (cat#200-092-156, Jackson ImmunoResearch Laboratories, Inc., West. Grove, PA, USA) at 1:500 dilution and a rabbit anti-FITC (cat# 4510-7804, Bio-Rad Laboratories, Hercules, CA, USA) at 1:1000 dilution + 2% normal mouse serum. The TdT-enzymes were the following: 1 mL antibody-diluent (cat#AR9352, Leica Microsystems Inc., Newcastle, UK), 100 µL TdT-buffer ((TUNEL-Box), cat# M1893, Promega, Madison, WI, USA), 1 µL dUTP (TUNEL-Box) DIG-11-dUTP (cat#11 570013 910, Roche Diagnostics International Ltd, Rotkreuz, Switzerland), 4 µL TdT-enzyme ((TUNEL-Box), cat#M1875, Promega, Madison, WI, USA). The cytoplasm of apoptotic cells contained red granules and the number of apoptotic cells in 10 random high-power fields (×400) was calculated. The apoptotic index was expressed as the number of apoptotic cells/100 cells (%).

### 2.9. Statistical Method

Results are expressed as mean and standard deviation (SD). For cytokines analysis the median and interquartile range (IQR) were used as measures of central tendency and dispersion, respectively. A nonparametric Mann–Whitney U-test was used for non-continuous data. Data with a time component were compared using 2-way analysis of variance (ANOVA). Statistical analysis was performed with GraphPad Prism version 8 software (GraphPad Software, Inc., La Jolla, CA, USA). Differences were considered significant at *p* < 0.05.

## 3. Results

### 3.1. An In Vitro Assay Determined the Non-Toxic Concentrations of K(ATP) Modulators for Use during EVLP

**Figure 1:**
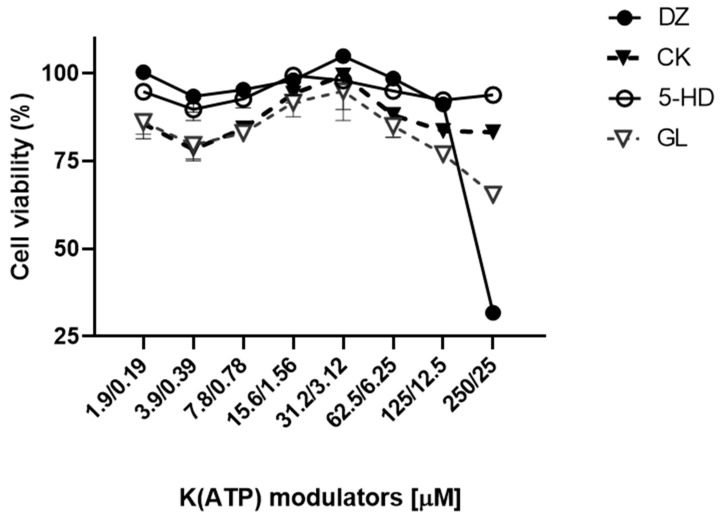
MTT assay with the rat IL2 lung epithelial cells exposed for 24 h to increasing concentrations of the different K(ATP) channel modulators. We performed two-fold dilution of the K(ATP) channel modulators with starting concentration of 250 µM for DZ, 5-HD, and GL or 25 µM for CK. Average results of three experiments with % CV.

As a starting point before applying a defined pharmacological concentration of K(ATP) channel modulators to the lung tissues during EVLP experiments, we searched the literature for different K(ATP) channel modulators and their previously nontoxic concentration used in vivo and in vitro. We then designed, with the rat IL2 lung epithelial cell line as a target, the in vitro MTT viability assay based on a 24 h incubation with increasing concentrations of the different K(ATP) channel modulators (Figure 1). From the result of this experiment, we selected the non-toxic concentration dosage for each of the four K(ATP) channel modulators for use during EVLP as follows: 100 µM for 5-HD, DZ, and GL, and 10 µM for CK.

### 3.2. Lung Physiology during EVLP

**Figure 2:**
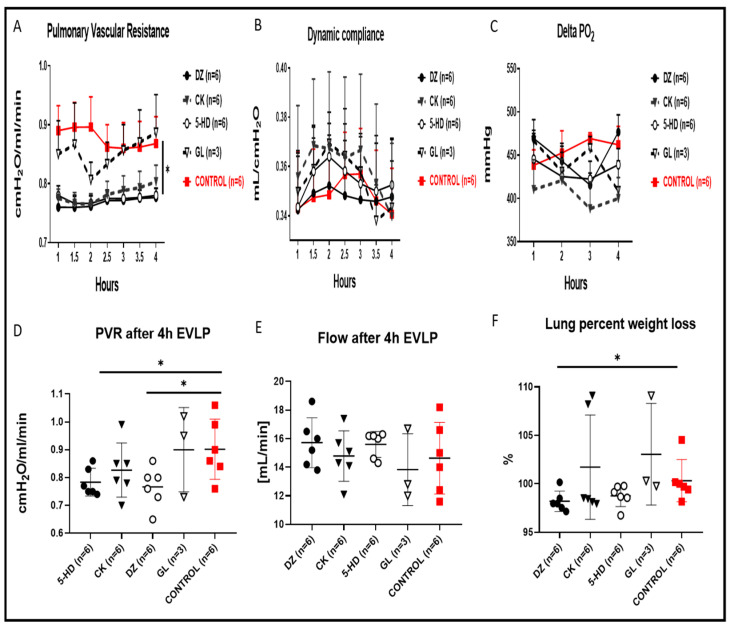
Lung PVR, Cdyn, oxygenation index, flow and percentage lung weight changes during the 4 h EVLP and following the end of the experimental EVLP stress test. (**A**) PVR in DZ and 5-HD was significantly lower than that of the control (* *p* < 0.05), whereas the PVR observed in the GL condition overlapped the control. (**B**) There were no significant differences in Cdyn between any K(ATP) channel modulators and the control. (**C**) There were no significant differences for the ΔpO_2_ between any K(ATP) channels modulators and the control. (**D**) PVR in 5-HD and DZ was significantly lower than that of the control (* *p* < 0.05), after the end of the EVLP stress test. (**E**) There were no significant differences in flow after 4 h of EVLP between any K(ATP) channels modulators and the control. (**F**) Percentage lung weight after 4 h of EVLP was significantly lower with the DZ condition as compared to the control (* *p* < 0.05), and lungs treated with 5-HD had a lower weight, but this was not significantly different when compared to the controls.

When compared to the control group, the PVR recorded in EVLP performed with DZ or 5-HD added to the perfusates was significantly lower (Figure 2A, * *p* < 0.05), and the PVR in the CK group was lower but not statistically different from the PVR of the control group. The GL condition overlapped the control and was the worst PVR value within all the K(ATP) channel modulators. During the end-of-EVLP stress test, the PVR for DZ and 5-HD (Figure 2D, * *p* < 0.05), but not the PVR of CK or GL, was also significantly lower than the control group. Concomitantly, during the end-of-EVLP stress test, we recorded higher flow for DZ, 5-HD, and GL (Figure 2E), but these flow values were not significantly different when compared to the control. Cdyn in 5-HD and DZ groups was comparable to that in the control group, although we observed slightly higher Cdyn at the last time point recorded for 5-HD and DZ (Figure 2B). Regarding ΔpO_2_, the DZ group had the highest ΔpO_2_ at the end of 4 h of EVLP, but we did not observe any statistical differences among any of the K(ATP) channel modulators (Figure 2C) when compared to the control. The rather high percentage lung weight gain at the end of EVLP in either of the two plasma membrane and/or sarcolemmal-specific forms of K(ATP) channel modulators GL or CK (Figure 2F) was probably the triggering cause for the lower ΔpO_2_ (Figure 2C) and higher PVR (Figure 2D) measured after 4 h of EVLP. Interestingly, we observed a significantly higher percentage lung weight loss in the DZ group after 4 h of EVLP (Figure 2F, * *p* < 0.05), and also a reduced edema formation in the 5-HD condition, but for 5-HD, this reduction was not significant when compared to the control group. Together, all these parameters point to a rather favorable outcome when the two mitochondria-specific modulators and especially the opener DZ are used in comparison to a worst outcome with the two plasma membrane and/or sarcolemmal-specific forms of K(ATP) channel modulators.

### 3.3. Lung Tissue Biochemical Measurements

**Figure 3:**
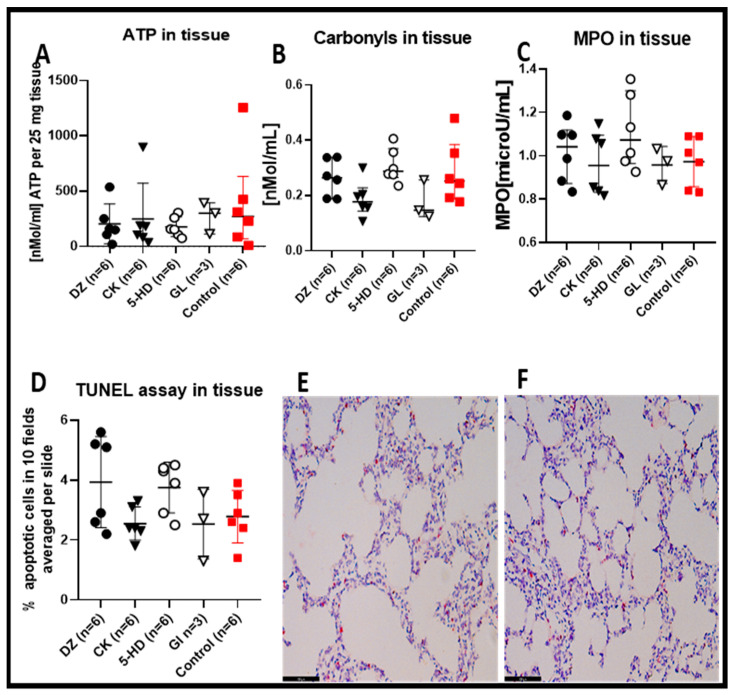
Tissue biochemical measurement. (**A**) There were no statistical differences in tissue ATP content in the perfused lungs after 4 h EVLP between the control and the 4 types of K(ATP) channels modulators. (**B**) The carbonyl proteins detected in the lung exposed to CK and GL K(ATP) channel modulators were lower, although not significantly when compared to the control. (**C**) The MPO in the lung exposed to DZ and 5-HD K(ATP) channel modulators did not have statistically significantly higher values versus the control. (**D**) TUNEL-positive cells detected in the lung tissues exposed to the different K(ATP) channel modulators were not significantly different compared to control. Representative TUNEL-stained sections for assessment of lung apoptotic cells in the DZ group (**E**) and the control group (**F**). Sections are shown at ×200 magnification at the end of 4 h EVLP. The scale bar indicates 100 µm length.

After 4 h of EVLP, there were no statistical differences regarding the tissue ATP content among the lungs treated with any of the four K(ATP) channel modulators or the control (Figure 3A). For all the K(ATP) channel modulators, the tissue content of carbonylated proteins (Figure 3B), MPO (Figure 3C) and the TUNEL staining (Figure 3D–F) were comparable to the control group. Interestingly, for all these parameters, the mitochondria-specific modulators DZ and the 5-HD had comparatively higher average values for carbonylated proteins, for MPO, for the TUNEL analysis (see also histological staining (Figure 3E,F)) and lower ATP values compared to the two plasma membrane and/or sarcolemmal-specific forms of K(ATP) channel modulators CK and GL.

### 3.4. Perfusate Cytokines, Chemokines and Mediator of Wound Healing

**Figure 4:**
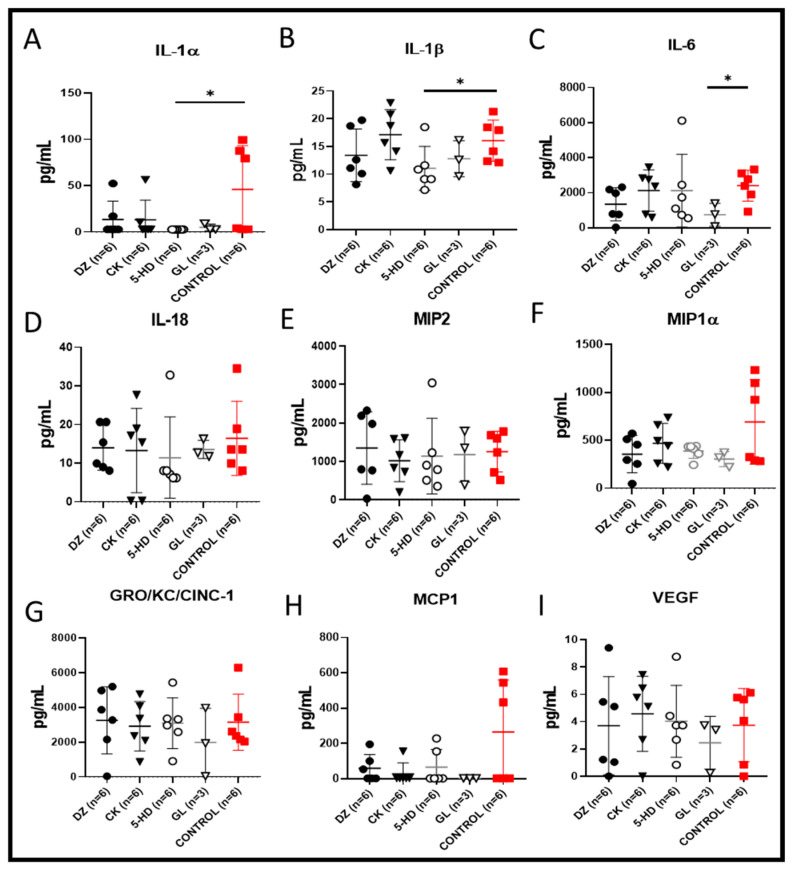
Perfusate concentration of cytokines, chemokines and mediator of wound healing after 4 h of EVLP. (**A**) Interleukin-1 beta (IL-1β) and (**B**) interleukin-1 alpha (IL-1α) were significantly lower with 5-HD (* *p* < 0.05), whereas (**C**) IL-6 was significantly lower with GL when compared to the control (* *p* < 0.05). (**D**) IL18, (**E**) MIP2, (**F**) MIP-1α, (**G**) GRO/KC/CINC-1, (**H**) MCP-1, and (**I**) VEGF were reduced in the presence of the channel blockers 5-HD and GL, but these levels were not significantly lower than the control value.

In the presence of 5-HD, the cytokines interleukin-1β (IL-1β) and interleukin-1α (IL-1α) were significantly lower when compared to the level of those cytokines observed in the control (* *p* < 0.05, respectively, Figure 4A,B). In the GL group, the level of interleukin-6 (IL-6) was significantly lower when compared to the level observed in the control (Figure 4C, * *p* < 0.05). These data demonstrate that the two K(ATP) channel openers significantly reduced the inflammatory status of the perfusate. In the presence of the K(ATP) channel blockers 5-HD and GL, interleukin-18 (IL-18), the monocyte chemoattractant protein 1 (MCP-1), macrophage inflammatory protein-2 (MIP2), macrophage inflammatory protein-1α (MIP-1α), GRO/KC/CINC-1 and the mediator of wound healing VEGF were reduced, but these differences were not statistically significant compared the control value (Figure 4D–I).

### 3.5. Correlations among Perfusate Cytokines and Tissue Carbonylated Proteins with the Four K(ATP) Channel Modulators or the Control

Spearman’s correlation analysis of the perfusate cytokines with the tissue carbonylated proteins did not show statistically significant difference at the end of EVLP for the four K(ATP) channel modulators or the control (Table 1). In the DZ group, and despite not reaching a significant correlation value, the protein carbonylation was showed a negative correlation trend for both IL-1β and MIP2, whereas in the control group, the protein carbonylation and IL-6 were positively correlated.

### 3.6. Biochemical Measurements in the Perfusate during EVLP

**Figure 5:**
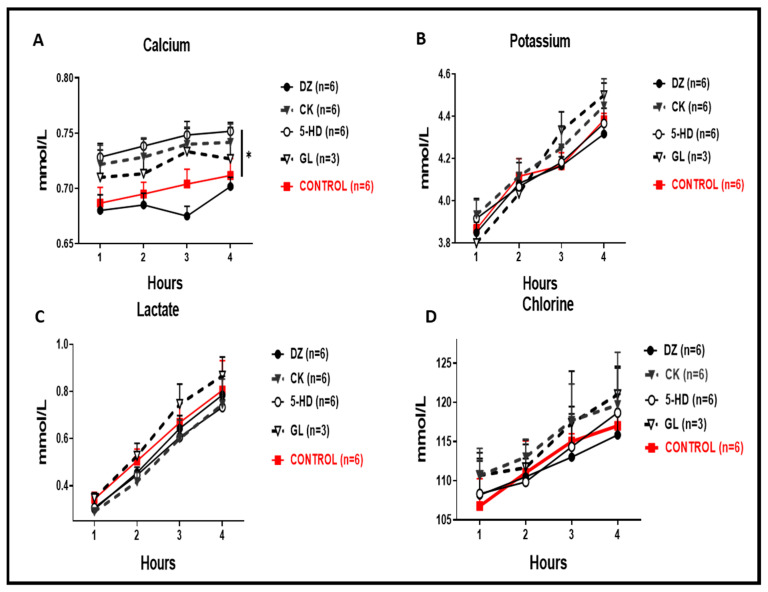
Clinical biochemistry of the perfusate. (**A**) Calcium perfusate concentrations were significantly higher in 5-HD and CK compared to the control (* *p* < 0.05). (**B**) Concentrations of potassium in the perfusate were not significantly different in any condition compared to the control. (**C**) Lactate levels in all conditions were comparable to the control. (**D**) Chlorine levels in all conditions were comparable to the control.

In the perfusate, we recorded significantly higher calcium concentrations for 5-HD and CK when compared to the control (Figure 5A, * *p* < 0.05), whereas for DZ we recorded a lower calcium concentration, but this was not significantly lower when compared to the control. Perfusate concentrations of potassium, chlorine and lactate were also always reduced for the DZ group (Figure 5B–D).

## 4. Discussion

Lung transplantation (LTx) is the only viable treatment option for end-stage lung diseases. Nonetheless, in the search for a successful long-term outcome, an important obstacle to overcome is a severe form of I/R lung injury that leads to lung graft failure [21]. It is assumed that pharmacological modulation of K(ATP) could be a good target to protect the lung graft prior to transplantation with corresponding improvement in the clinical outcome [13], but the effect of K(ATP) channel openers or blockers during ischemia is still controversial in many organs and tissues [17,22,23,24,25].

We sought to inhibit the membrane hyperpolarization during reperfusion using a variety of K(ATP) channel modulators and designed our experiments with the blockers used as a control for the experiment performed with openers and selected modulators directed against either the mitochondrial-specific K(ATP) channels (DZ as an opener and 5-HD as a blocker) or the plasma membrane and/or sarcolemmal-specific forms of the K(ATP) channel (CK as an opener and GL as a blocker). Only few publications report the use of K(ATP) channel modulators in the context of I/R injury in lung transplantation [26,27], but DZ injected intraperitoneally prior to lung transplantation in a rat recipient provided protective effects in an in vivo I/R model [26]. In our EVLP experiments, the presence of the two mitochondrial-specific K(ATP) channel modulators DZ and 5-HD resulted in significantly lower PVR and importantly, the lung weight loss was significantly higher for DZ, whereas the use of two plasma membrane and/or sarcolemmal-specific K(ATP) modulators did not favorably influence these two parameters when compared to the control group. In the lung endothelium, when the blood flow is stopped, a mechanosignaling cascade is initiated which continues during the cold ischemic preservation time of the graft and when the flow is restored (reperfusion period). As a consequence of this triggered mechanosignaling cascade, there is a sodium influx and persistent cell membrane depolarization, which is characterized by lowered intracellular potassium [10]. Accordingly, the enhancement of calcium-sensitive potassium channels and of the K(ATP) channel during lung graft ischemia would provide protection through an antagonizing effect on membrane depolarization (i.e., favoring hyperpolarization). As both the liver and kidneys are absent from acellular organ machine perfusion protocols, the circulating concentration of potassium, sodium, chlorine and lactate increases as the pH drops. In our EVLP experiments with 5-HD, we recorded significantly more calcium ions in comparison to DZ, and the presence of 5-HD gave rise to a not significantly higher amount of potassium, chlorine and sodium ions in the EVLP perfusates. Calcium acts as one of the primary regulators of osmotic stress, but we did not observe any lung weight gain with 5-HD. The hypercalcemia and hyperkalemia are possibly linked to the cell death-related release of intracellular calcium/potassium into the perfusate and the excessive ion entry may have damaged the lung parenchyma which underwent cell apoptosis or cell death by necrosis. Nonetheless, neither 5-HD nor DZ showed significant higher TUNEL count of apoptotic cells. Moreover, the use of K(ATP) channel modulators during lung graft ischemia would provide membrane hyperpolarization and protection by attenuating ROS production [28], leading to the abortion of inflammasome priming and activation, and accordingly muting the release of pro-inflammatory cytokines [14,29]. In our EVLP experiments, it is in the perfusate of the 5-HD EVLP group and after 4-hour EVLP that we detected a significantly reduced amount of the pro-inflammatory cytokines IL1α and IL1β. Some improvement in the lung inflammatory status during EVLP was also observed with the plasma membrane and/or sarcolemmal-specific K(ATP) modulators under GL conditions with a significantly lower amount of the pro-inflammatory IL-6 when compared to the control. GL inhibits inflammasome activation, preventing the processing of IL1β and IL-18, and consequently decreases the release of IL-6 [12,14]. It is therefore possible that GL may be an effective treatment in preventing both the inflammation of the endothelium and the recruitment of leukocytes following reperfusion after transplantation [30,31]. 

Nonetheless, the use of K(ATP) channel modulators for I/R injury is controversial. Studies on renal I/R injury show that K(ATP) channel openers protected against renal I/R injury [32]; however, other studies reported that K(ATP) channels blockers prevented renal I/R injury [30]. In comparison to the EVLP experiments presented in this publication, these organ clamping models do not describe the distinct damage of the cold ischemia and the long loss of flow typically encountered in organ transplantation. Usually, lung experiments described in the literature either modulated the K(ATP) channels in the context of a whole animal [26,27], or for pre- or post-conditioning purposes [33,34], but with EVLP and perfusate, we do not have these confounding factors found in a whole animal, and we consider that the modulation of the K(ATP) channels in a DCD lung donor with the EVLP approach is a good experimental system to test I/R injury. One additional confounding factor may have been the fact that DZ and 5-HD were reported to have other cellular effects than the modulation of the K(ATP) channels in rat and pig kidney cell lines [35] or in the pig heart [36]. DZ is also known to produce multiple side effects because of its interactions with different cellular targets such as the plasma membrane and/or sarcolemmal-specific forms of K(ATP) channels, F0F1 ATP synthase and succinate dehydrogenase [37]. As reported in heart and liver mitochondria [38,39], 5-HD has also displayed off-target effects other than mitochondrial K(ATP) modulation and showed a lack of activity in inhibiting oxygen-consuming mitochondria derived from the skeletal muscle [39,40,41]. Additionally, regarding DZ and 5HD, the hypothetical and controversial presence of mitochondrial-specific K(ATP) channels was only recently identified [28,29,30]. The constituents of this mitochondrial K(ATP) channel are MITOK and MISUR, which functionally transport K^+^ across the inner mitochondrial membrane [31,32,33]. It is also probably due to the confounding GL mode of action on molecules such as thromboxane A2 receptor antagonism [42] and by affecting the Na^+^/K^+^-ATPase inhibition more than 5-HD [43] that GL may have shown very unfavorable outcomes in our EVLP experiments.

The results recorded with the two plasma membrane and/or sarcolemmal-specific channels modulators already used in treating organs before transplantation, for both the control of I/R injury and the control of ROS generation, are less convincing than the parameters recorded with the mitochondrial-specific K(ATP) channels modulators. We observed in the EVLP experiments of the GL and CK groups an enhancement of many important physiological negative aspects when compared to the control group, such as an increase in the weight of the lung due to edema or unimproved perfusate flow. Moreover, we detected the lactate production as being higher for GL and lower for 5-HD, and in both CK and GL, we observed not significantly higher concentrations of potassium and chlorine ions, whereas calcium was significantly lower in the CK group. The clinical relevance of modulation of membrane depolarization is to reduce the generation of ROS [10]. The generation of ROS is particularly toxic for the mitochondria, and the advantage of using pharmaceutical approaches which modulate membrane depolarization such as specific K(ATP) channel modulators is that these channels are present in the mitochondria and in the plasma membrane. Interventional strategies with K(ATP) channel modulators could reduce damage during the storage of donor lungs, with the goal of increasing the availability of lungs for transplantation and thus improving access to lung transplantation. This interventional strategies with K(ATP) channel modulators also have clinical relevance for conditions other than lung harvesting/storage during lung transplantation, and could be used for pulmonary embolism and lung bypass surgery.

To summarize our findings, we identified a potential use of mitochondrial-specific K(ATP) channel opener DZ. The DZ may counteract both the damage initiated by the early mechanosignaling cascade and the long loss of flow encountered in organ transplantation. DZ may have counterbalanced the cell membrane depolarization due to inactive K(ATP) channels and may have tempered the early onset of ROS-producing enzymes and the increased NADPH oxidase activity, leading to ischemia reperfusion injuries.

## Figures and Tables

**Table 1:** Correlation between tissue carbonyl protein contents and perfusate cytokine levels.

Conditions	DZ	CK	5-HD	GL	Control
	r	r	r	r	r
	*(p)*	*(p)*	*(p)*	*(p)*	*(p)*
IL-1α	−0.02857	−0.4286	−0.4285	0.5	0.0285
	*(1)*	*(0.4194)*	*(0.4194)*	*(1)*	*(1)*
IL-1β	−0.7728	−0.0857	−0.2029	0.5	0.0285
	*(0.1028)*	*(0.9194)*	*(0.7139)*	*(1)*	*(1)*
IL-6	−0.2571	−0.3143	0.2571	0.5	0.4286
	*(0.6583)*	*(0.5639)*	*(0.6583)*	*(1)*	*(0.4194)*
IL-18	−0.4928	−0.0289	−0.5296	−0.7697	−0.0289
	*(0.3556)*	*(1)*	*(0.2972)*	*(0.4408)*	*(1)*
MIP2	−0.7714	0.2571	−01429	0.5	−0.2
	*(0.1028)*	*(0.6583)*	*(0.8028)*	*(1)*	*(0.7139)*
MIP-1α	0.0285	−0.4286	0.6571	0.5	−0.0857
	*(1)*	*(0.4194)*	*(0.1750)*	*(1)*	*(0.9194)*
GRO/KC	−0.4286	0.3143	−0.4286	0.5	0.0285
	*(0.4194)*	*(0.5639)*	*(0.4194)*	*(1)*	*(1)*
MCP-1	0.0285	0.2571	0.3339	0.5	−0.08571
	*(1)*	*(0.6583)*	*(0.4972)*	*(1)*	*(0.9194)*
VEGF	0.08571	−0.1429	−0.2029	1	0.2571
	*(0.9194)*	*(0.8028)*	*(0.7139)*	*(0.3333)*	*(0.6584)*

Analyzed by Spearman’s correlation analysis.

## Data Availability

The data sets generated during and/or analyzed during the current study are available from the corresponding author on reasonable request.

## Abbreviations

5-HD	5-hydroxydecanoic acid.
K(ATP)	ATP sensitive potassium channel
Cdyn	Dynamic compliance
CK	cromakalim
DMSO	dimethyl sulfoxide
DCD	donation after circulatory death
DZ	diazoxide
EVLP	ex vivo lung perfusion
FiO2	inspired oxygen fraction
GL	glybenclamide
I/R	ischemia-reperfusion
MPO	myeloperoxidase
MTT	3-(4,5-dethylthiazol-2-yl)-2,5-diphenyltetrazolium bromide
PAP	pulmonary arterial pressure
PVR	pulmonary vascular resistance
ROS	reactive oxygen species
TUNEL	terminal deoxynucleotidyl transferase dUTP nick end labelling
